# Crystal structure and Hirshfeld surface analysis of (2*E*)-1-(3-bromo­phen­yl)-3-(4-fluoro­phen­yl)prop-2-en-1-one

**DOI:** 10.1107/S2056989018018418

**Published:** 2019-01-04

**Authors:** Zeliha Atioğlu, S. Bindya, Mehmet Akkurt, C. S. Chidan Kumar

**Affiliations:** a İlke Education and Health Foundation, Cappadocia University, Cappadocia Vocational College, The Medical Imaging Techniques Program, 50420 Mustafapaşa, Ürgüp, Nevşehir, Turkey; b Department of Chemistry, Sri Jayachamarajendra College of Engineering, JSS Science & Technology University, Mysore 570006, Karnataka, India; c Department of Physics, Faculty of Sciences, Erciyes University, 38039 Kayseri, Turkey; d Department of Engineering Chemistry, Vidya Vikas Institute of Engineering & Technology, Visvesvaraya Technological University, Alanahalli, Mysuru 570028, Karnataka, India

**Keywords:** crystal structure, 3-bromo­phenyl ring, 4-fluoro­phenyl ring, a prop-2-en-1-one spacer, Hirshfeld surface analysis

## Abstract

In the title compound, C_15_H_10_BrFO, the mol­ecular structure consists of a 3-bromo­phenyl ring and a 4-fluoro­phenyl ring linked *via* a prop-2-en-1-one spacer. The 3-bromophenyl and 4-fluorophenyl rings make a dihedral angle of 48.90 (15)°. The mol­ecule has an *E* configuration about the C=C bond and the carbonyl group is *syn* with respect to the C=C bond. In the crystal, mol­ecules are linked by C—H⋯π inter­actions between the bromo­phenyl and fluoro­phenyl rings of mol­ecules, resulting in a two-dimensional layered structure parallel to the *ab* plane. The mol­ecular packing is stabilized by weak Br⋯H and F⋯H contacts, one of which is on the one side of each layer, and the second is on the other. The inter­molecular inter­actions in the crystal packing were further analysed using Hirshfeld surface analysis, which indicates that the most significant contacts are Cl⋯H/H⋯Cl (20.8%), followed by C⋯H/H⋯C (31.1%), H⋯H (21.7%), Br⋯H/H⋯Br (14.2%), F⋯H/H⋯F (9.8%), O⋯H/H⋯O (9.7%).

## Abstract 2

In the title compound, the 3-bromo­phenyl and 4-fluoro­phenyl rings, linked *via* a prop-2-en-1-one spacer, make a dihedral angle of 48.90 (15)°. In the crystal, mol­ecules are linked by C—H⋯π inter­actions between the bromo­phenyl and fluoro­phenyl rings of mol­ecules, resulting in a two-dimensional layered structure parallel to the *ab* plane.

## Chemical context   

An aromatic ketone and an enone that forms the central core for a variety of important biological compounds, which are known collectively as chalcones or chalconoids. Chalcones are 1,3-diphenyl-2-propene-1-one, in which two aromatic rings are linked by a three carbon α,β-unsaturated carbonyl system. The α,β-unsaturated ketone group in chalcones is responsible for their enzyme inhibitory activity including xanthine oxidase, aldose reductase, soluble epoxide hydro­lase, protein tyrosine kinase, quinonone reductase and mono amine oxidase (Amita *et al.*, 2014 ▸). Chalcones are abundant in nature starting from ferns to higher plants and a number of them are polyhy­droxy­lated in the aryl rings. They are considered to be precursors of flavonoids and isoflavonoids. Chalcones possess conjugated double bonds and a completely delocalized π-electron system on both benzene rings. Mol­ecules that possess such a system have relatively low redox potentials and have a greater probability of undergoing electron-transfer reactions. Crystal structures have been reported for 3-(3-bromo­phen­yl)-1-(4-bromo­phen­yl)prop-2-en-1-one (Teh *et al.*, 2006 ▸), 3-(3-bromo­phen­yl)-1-(2-naphth­yl)prop-2-en-1-one (Moorthi *et al.*, 2007 ▸), (*E*)-1-(3-bromo­phen­yl)-3-(4-eth­oxy­phen­yl)prop-2-en-1-one (Fun *et al.*, 2008 ▸), (*E*)-3-(biphenyl-4-yl)-1-(3-bromo­phen­yl)prop-2-en-1-one (Dutkiewicz *et al.*, 2009 ▸), (2*E*)-1-(3-bromo­phen­yl)-3-(6-meth­oxy-2-naphth­yl)prop-2-en-1-one (Harrison *et al.*, 2010 ▸), (2*E*)-1-(3-bromo­phen­yl)-3-(4,5-dimeth­oxy-2-nitro­phen­yl)prop-2-en-1-one (Jasinski *et al.*, 2010 ▸), (*E*)-1-(3-bromo­phen­yl)-3-(3,4-di­meth­oxy­phen­yl)prop-2-en-1-one (Escobar *et al.*, 2012 ▸), (*E*)-1-(3-bromo­phen­yl)-3-(4-nitro­phen­yl)prop-2-en-1-one (Harini *et al.*, 2017 ▸) and (*E*)-1-(3-bromo­phen­yl)-3-(3-fluoro­phen­yl)prop-2-en-1-one (Rajendraprasad *et al.*, 2017 ▸). We herewith report the crystal and mol­ecular structure of the title compound.
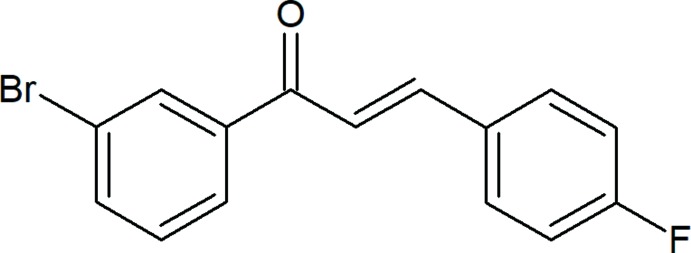


## Structural commentary   

**Figure 1:**
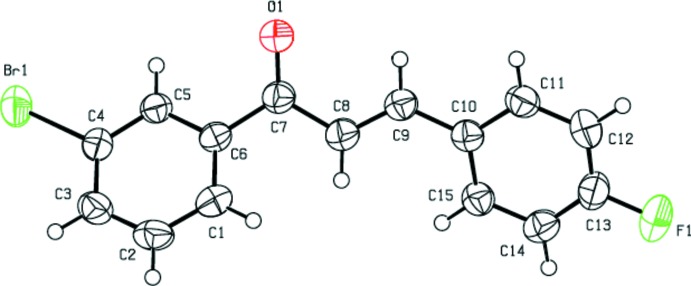
The mol­ecular structure of the title compound, showing the atom labelling and displacement ellipsoids drawn at the 50% probability level.

As shown in Fig. 1 ▸, the title compound is constructed from two aromatic rings (3-bromo­phenyl and 4-fluoro­phenyl rings), which are linked by a C=C—C(=O)—C enone bridge. Probably as a result of the steric repulsion between the fluoride and bromine atoms of adjacent mol­ecules, the C5—C6—C7— O1 and O1—C7—C8—C9 torsion angles about the enone bridge are 25.1 (4) and 14.0 (5) °, respectively. Hence, the dihedral angle between the 3-bromo­phenyl ring and the 4-fluoro­phenyl ring increases to 48.90 (15)°. The mol­ecular conformation of the title compound is stabilized by intra­molecular C—H⋯Cl contacts (Table 1 ▸), producing *S*(6) and *S*(5) ring motifs. The bond lengths and angles are comparable with those found in the related compounds (2*E*)-3-(3-chloro­phen­yl)-1-(3,4-di­meth­oxy­phen­yl)-prop-2-en-1-one (Sheshadri *et al.*, 2018*a* ▸), (2*E*)-3-(3-bromo-4-fluoro­phen­yl)-1-(3,4-di­meth­oxy­phen­yl)prop-2-en-1-one (Sheshadri *et al.*, 2018*b* ▸), (*E*)-3-(3,4-di­meth­oxy­phen­yl)-1-(1-hy­droxy­naphthalen-2­yl)prop-2-en-1-one (Ezhilarasi *et al.*, 2015 ▸), (*E*)-1-(3-bromo­phen­yl)-3-(3,4-di­meth­oxyphen­yl)prop-2-en-1-one (Escobar *et al.*, 2012 ▸) and (*E*)-3-(2-bromo­phen­yl)-1-(3,4-di­meth­oxy­phen­yl)prop-2-en-1-one (Li *et al.*, 2012 ▸).

## Supra­molecular features and Hirshfeld surface analysis   

**Figure 2:**
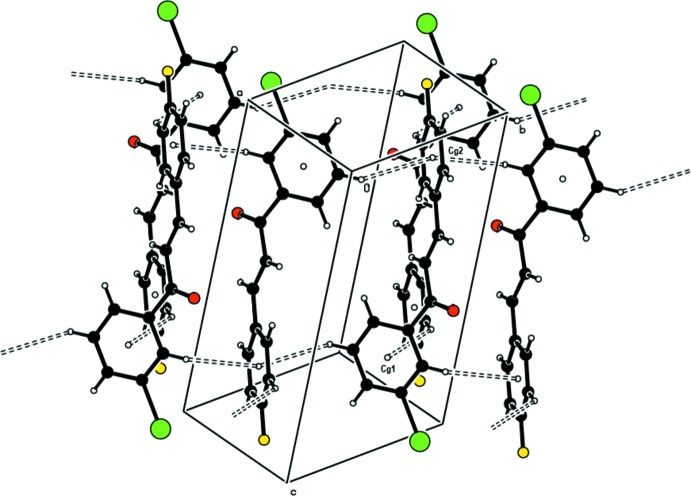
A view of the C—H⋯π inter­actions in the title compound.

In the crystal, mol­ecules are linked by C—H⋯π inter­actions between the bromo­phenyl and fluoro­phenyl rings of mol­ecules, resulting in a two-dimensional layered structure parallel to the *ab* plane (Table 1 ▸; Fig. 2 ▸). The mol­ecular packing is stabilized by weak Br⋯H and ⋯H contacts, one of which is on the one side of a layer, and the second is on the other. A summary of the short contacts is given in Table 2 ▸.

Hirshfeld surfaces and fingerprint plots were generated for the title compound using *CrystalExplorer* (McKinnon *et al.*, 2007 ▸). Hirshfeld surfaces enable the visualization of inter­molecular inter­actions by different colours and colour intensity, representing short or long contacts and indicating the relative strength of the inter­actions.

**Figure 3:**
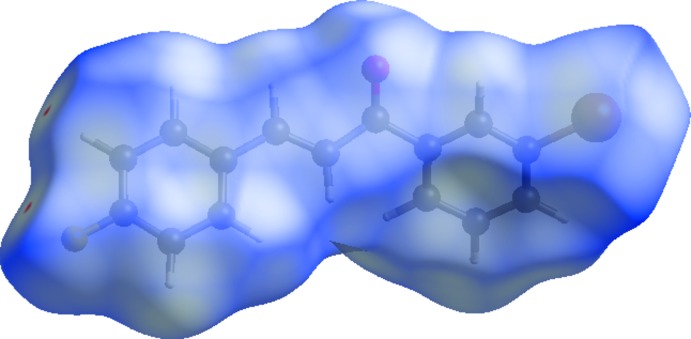
A view of the Hirshfeld surface of the title compound mapped over *d*_norm_, using a fixed colour scale of −0.0186 (red) to 1.3784 (blue) a.u.

The function *d*_norm_ is a ratio enclosing the distances of any surface point to the nearest inter­ior (*d*_i_) and exterior (*d*_e_) atom and the van der Waals radii of the atoms (Hirshfeld, 1977 ▸; Soman *et al.*, 2014 ▸). The function *d*_norm_ will be equal to zero when inter­molecular distances are close to van der Waals contacts. They are indicated by a white colour on the Hirshfeld surface, while contacts longer than the sum of van der Waals radii with positive *d*_norm_ values are coloured in blue. The surface plot for *d*_norm_ (Fig. 3 ▸) was generated using a high standard surface resolution over a colour scale of −0.0186 to 1.3784 a.u.

**Figure 4:**
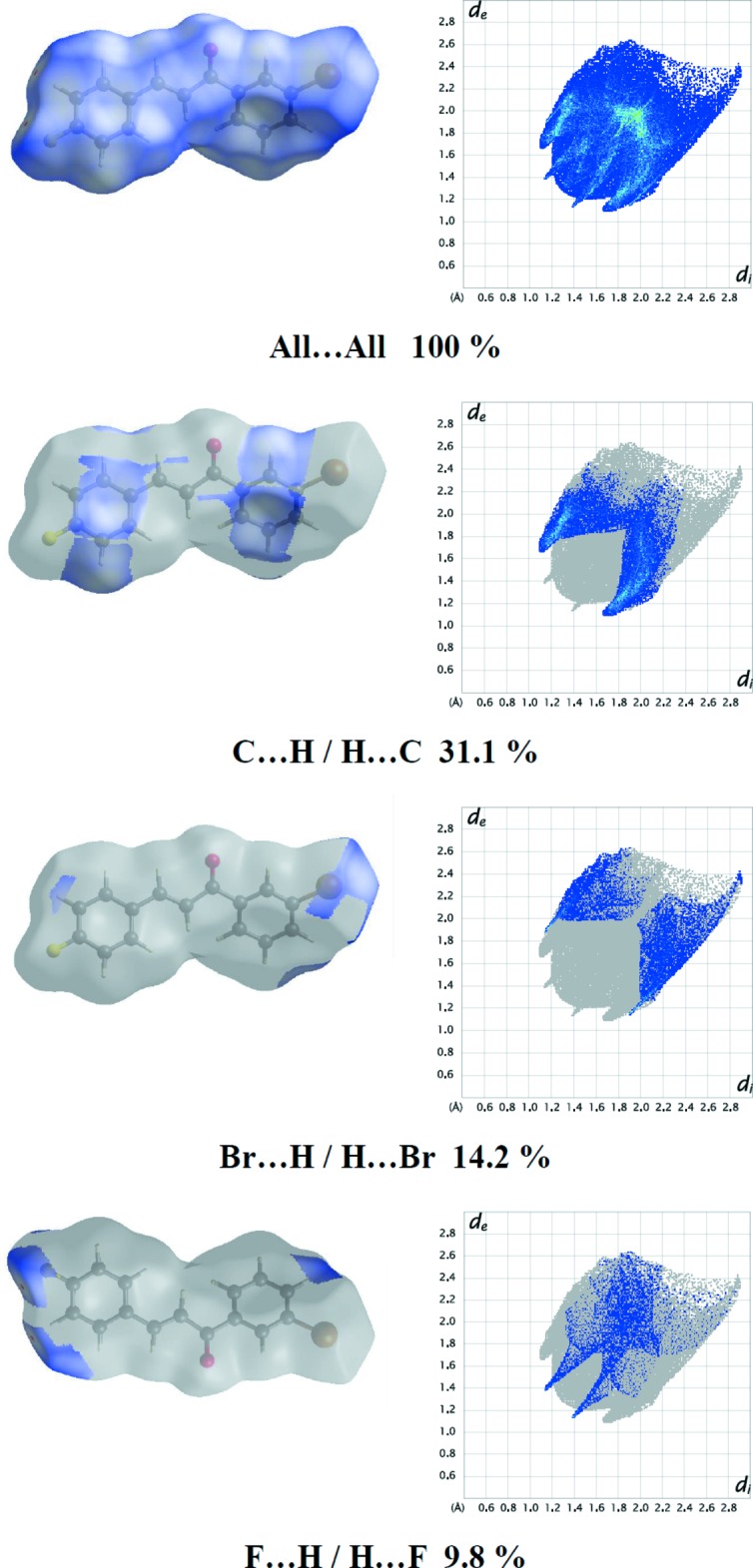
The two-dimensional fingerprint plots of the title compound.

The overall two-dimensional fingerprint plot for the title compound and those delineated into C⋯H/H⋯C, H⋯H, Br⋯H/H⋯Br, F⋯H/H⋯F and O⋯H/H⋯O contacts are illustrated in Fig. 4 ▸. The percentage contributions of the various inter­atomic contacts to the Hirshfeld surfaces are given in Table 3 ▸. The presence of C—H⋯π inter­actions in the crystal is indicated by the pair of characteristic wings in the fingerprint plot delineated into C⋯H/H⋯C contacts (31.1% contribution to the Hirshfeld surface). The C⋯H/H⋯C inter­actions are represented by the spikes at the bottom right and left (*d*_e_ + *d*_i_ ≃ 2.75 Å). H⋯H contacts are disfavoured when the number of H atoms on the mol­ecular surface is large. The Br⋯H/H⋯Br and F⋯H/H⋯F contacts (Fig. 4 ▸) in the structure with 14.2 and 9.8% contributions, respectively, to the Hirshfeld surface are viewed as pairs of spikes with the tips at *d*_e_ + *d*_i_ ≃ 3.05 and 2.45 Å, respectively.

## Synthesis and crystallization   

The title compound was synthesized as per the procedure reported earlier (Kumar *et al.*, 2013*a* ▸,*b* ▸). 1-(3-Bromo­phen­yl)ethanone (0.01 mol) and 4-fluoro­benzaldehyde (0.01 mol) were dissolved in 30 ml methanol. A catalytic amount of NaOH was added to the solution dropwise under vigorous stirring. The reaction mixture was stirred for about 4 h at room temperature. The formed crude products were filtered, washed successively with distilled water and recrystallized from methanol to obtain the title chalcone. The melting point (338–342 K) was determined using a Stuart Scientific (UK) apparatus.

## Refinement   

Crystal data, data collection and structure refinement details are summarized in Table 4 ▸. H atoms were positioned geometrically and refined using riding model, with C—H = 0.93 Å and *U*_iso_(H) = 1.2*U*_eq_(C). Owing to poor agreement between observed and calculated intensities, thirteen outliers (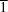
15, 131, 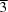
26, 043, 254, 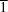
23, 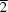
28, 150, 253, 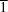
11, 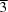
25, 543, 623) were omitted in the final cycles of refinement.

## Supplementary Material

Crystal structure: contains datablock(s) I. DOI: 10.1107/S2056989018018418/xu5955sup1.cif

Structure factors: contains datablock(s) I. DOI: 10.1107/S2056989018018418/xu5955Isup2.hkl

Supporting information file. DOI: 10.1107/S2056989018018418/xu5955Isup3.cml

CCDC reference: 1036740

Additional supporting information:  crystallographic information; 3D view; checkCIF report

## Figures and Tables

**Table 1:** Hydrogen-bond geometry (Å, °) *Cg*1 and *Cg*2 are the centroids of the 3-bromo­phenyl (C1–C6) and 4-fluoro­phenyl (C10–C15) rings, respectively.

*D*—H⋯*A*	*D*—H	H⋯*A*	*D*⋯*A*	*D*—H⋯*A*
C2—H2*A*⋯*Cg*2^i^	0.93	2.93	3.571 (4)	127
C5—H5*A*⋯*Cg*2^ii^	0.93	2.98	3.642 (3)	129
C14—H14*A*⋯*Cg*1^iii^	0.93	2.90	3.590 (3)	132

Symmetry codes: (i) 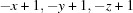; (ii) 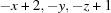; (iii) 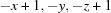.

**Table 2:** Summary of short inter­atomic contacts (Å) in the title compound

Contact	Distance	Symmetry operation
Br1⋯Br1	3.7222 (6)	3 − *x*, 1 − *y*, −*z*
H3*A*⋯Br1	3.19	2 − *x*, 1 − *y*, −*z*
H12*A*⋯F1	2.66	1 − *x*, −*y*, 2 − *z*
H1*A*⋯O1	2.82	−1 + *x*, *y*, *z*
H11*A*⋯C3	3.01	2 − *x*, 1 − *y*, 1 − *z*
H14*A*⋯C6	2.95	1 − *x*, −*y*, 1 − *z*
H2*A*⋯C10	2.89	1 − *x*, 1 − *y*, 1 − *z*
H5*A*⋯C15	3.00	2 − *x*, −*y*, 1 − *z*

**Table 3:** Percentage contributions of inter­atomic contacts to the Hirshfeld surface for the compound

Contact	Percentage contribution
C⋯H/H⋯C	31.1
H⋯H	21.7
Br⋯H/H⋯Br	14.2
F⋯H/H⋯F	9.8
O⋯H/H⋯O	9.7
C⋯C	3.4
Br⋯F/F⋯Br	3.1
F⋯C/C⋯F	1.8
Br.·C/C⋯Br	1.5
C⋯O/O⋯C	1.5
F⋯F	1.3
Br⋯Br	0.9

**Table 4:** Experimental details

Crystal data	
Chemical formula	C_15_H_10_BrFO
*M*_r_	305.14
Crystal system, space group	Triclinic, *P* 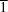
Temperature (K)	294
*a*, *b*, *c* (Å)	5.9255 (6), 7.5867 (8), 14.1427 (15)
α, β, γ (°)	89.774 (2), 82.671 (2), 87.712 (2)
*V* (Å^3^)	630.09 (11)
*Z*	2
Radiation type	Mo *K*α
μ (mm^−1^)	3.26
Crystal size (mm)	0.30 × 0.28 × 0.26

Data collection	
Diffractometer	Bruker APEXII CCD
Absorption correction	Multi-scan (*SADABS*; Bruker, 2007 ▸)
*T*_min_, *T*_max_	0.398, 0.431
No. of measured, independent and observed [*I* > 2σ(*I*)] reflections	9618, 2442, 2101
*R*_int_	0.023
(sin θ/λ)_max_ (Å^−1^)	0.617

Refinement	
*R*[*F* ^2^ > 2σ(*F* ^2^)], *wR*(*F* ^2^), *S*	0.038, 0.105, 1.10
No. of reflections	2442
No. of parameters	163
H-atom treatment	H-atom parameters constrained
Δρ_max_, Δρ_min_ (e Å^−3^)	0.76, −0.40

Computer programs: *APEX2* and *SAINT* (Bruker, 2007 ▸), *SHELXS97* (Sheldrick, 2008 ▸), *SHELXL2014* (Sheldrick, 2015 ▸), *ORTEP-3 for Windows* (Farrugia, 2012 ▸) and *PLATON* (Spek, 2009 ▸).

## supplementary crystallographic information

### Crystal data

**Table:**

C_15_H_10_BrFO	*Z* = 2
*M**_r_* = 305.14	*F*(000) = 304
Triclinic, *P*1	*D*_x_ = 1.608 Mg m^−^^3^
*a* = 5.9255 (6) Å	Mo *K*α radiation, λ = 0.71073 Å
*b* = 7.5867 (8) Å	Cell parameters from 4763 reflections
*c* = 14.1427 (15) Å	θ = 3.6–27.3°
α = 89.774 (2)°	µ = 3.26 mm^−^^1^
β = 82.671 (2)°	*T* = 294 K
γ = 87.712 (2)°	Block, colourless
*V* = 630.09 (11) Å^3^	0.30 × 0.28 × 0.26 mm

### Data collection

**Table:**

Bruker APEXII CCD diffractometer	2101 reflections with *I* > 2σ(*I*)
φ and ω scans	*R*_int_ = 0.023
Absorption correction: multi-scan (SADABS; Bruker, 2007)	θ_max_ = 26.0°, θ_min_ = 1.5°
*T*_min_ = 0.398, *T*_max_ = 0.431	*h* = −7→7
9618 measured reflections	*k* = −7→9
2442 independent reflections	*l* = −17→17

### Refinement

**Table:**

Refinement on *F*^2^	0 restraints
Least-squares matrix: full	Hydrogen site location: inferred from neighbouring sites
*R*[*F*^2^ > 2σ(*F*^2^)] = 0.038	H-atom parameters constrained
*wR*(*F*^2^) = 0.105	*w* = 1/[σ^2^(*F*_o_^2^) + (0.0481*P*)^2^ + 0.4984*P*] where *P* = (*F*_o_^2^ + 2*F*_c_^2^)/3
*S* = 1.10	(Δ/σ)_max_ = 0.001
2442 reflections	Δρ_max_ = 0.76 e Å^−^^3^
163 parameters	Δρ_min_ = −0.40 e Å^−^^3^

### Special details

**Table:**

Geometry. All esds (except the esd in the dihedral angle between two l.s. planes) are estimated using the full covariance matrix. The cell esds are taken into account individually in the estimation of esds in distances, angles and torsion angles; correlations between esds in cell parameters are only used when they are defined by crystal symmetry. An approximate (isotropic) treatment of cell esds is used for estimating esds involving l.s. planes.

### Fractional atomic coordinates and isotropic or equivalent isotropic displacement parameters (Å^2^)

**Table:**

	*x*	*y*	*z*	*U*_iso_*/*U*_eq_	
Br1	1.29286 (6)	0.37541 (6)	0.08193 (2)	0.06864 (18)	
F1	0.2394 (4)	−0.0245 (4)	0.91227 (16)	0.0855 (7)	
O1	1.2246 (4)	0.2758 (4)	0.46700 (17)	0.0609 (6)	
C1	0.7494 (5)	0.4234 (4)	0.3499 (2)	0.0485 (7)	
H1A	0.639101	0.432526	0.402871	0.058*	
C2	0.7020 (5)	0.4863 (5)	0.2621 (3)	0.0553 (8)	
H2A	0.560236	0.540073	0.257032	0.066*	
C3	0.8602 (5)	0.4709 (4)	0.1826 (2)	0.0530 (8)	
H3A	0.825976	0.511138	0.123703	0.064*	
C4	1.0720 (5)	0.3940 (4)	0.1920 (2)	0.0440 (6)	
C5	1.1280 (5)	0.3320 (4)	0.2783 (2)	0.0408 (6)	
H5A	1.271938	0.281871	0.283008	0.049*	
C6	0.9640 (5)	0.3463 (4)	0.3583 (2)	0.0401 (6)	
C7	1.0259 (5)	0.2821 (4)	0.4522 (2)	0.0453 (7)	
C8	0.8399 (5)	0.2254 (4)	0.5237 (2)	0.0491 (7)	
H8A	0.697720	0.207874	0.504874	0.059*	
C9	0.8697 (5)	0.1987 (4)	0.6141 (2)	0.0437 (6)	
H9A	1.012966	0.220989	0.630447	0.052*	
C10	0.7013 (5)	0.1380 (4)	0.6910 (2)	0.0402 (6)	
C11	0.7434 (5)	0.1542 (4)	0.7850 (2)	0.0479 (7)	
H11A	0.878507	0.202032	0.797670	0.058*	
C12	0.5888 (6)	0.1008 (5)	0.8598 (2)	0.0585 (8)	
H12A	0.617731	0.112494	0.922560	0.070*	
C13	0.3923 (6)	0.0304 (5)	0.8394 (2)	0.0550 (8)	
C14	0.3445 (5)	0.0080 (4)	0.7482 (2)	0.0500 (7)	
H14A	0.211180	−0.044035	0.736707	0.060*	
C15	0.4981 (5)	0.0640 (4)	0.6738 (2)	0.0456 (7)	
H15A	0.466180	0.052442	0.611505	0.055*	

### Atomic displacement parameters (Å^2^)

**Table:**

	*U*^11^	*U*^22^	*U*^33^	*U*^12^	*U*^13^	*U*^23^
Br1	0.0651 (3)	0.0932 (3)	0.0443 (2)	0.00443 (19)	0.00298 (15)	0.01381 (18)
F1	0.0780 (15)	0.117 (2)	0.0573 (13)	−0.0210 (14)	0.0140 (11)	0.0172 (13)
O1	0.0431 (12)	0.0916 (19)	0.0486 (13)	−0.0062 (12)	−0.0076 (9)	0.0087 (12)
C1	0.0376 (14)	0.0507 (18)	0.0559 (18)	−0.0022 (13)	−0.0009 (13)	−0.0040 (14)
C2	0.0405 (15)	0.0551 (19)	0.072 (2)	0.0047 (14)	−0.0157 (15)	0.0050 (16)
C3	0.0502 (17)	0.0548 (19)	0.0564 (19)	−0.0041 (14)	−0.0162 (14)	0.0139 (15)
C4	0.0426 (14)	0.0470 (16)	0.0416 (15)	−0.0025 (13)	−0.0023 (12)	0.0043 (12)
C5	0.0359 (13)	0.0421 (15)	0.0444 (15)	−0.0014 (12)	−0.0049 (11)	0.0028 (12)
C6	0.0368 (13)	0.0397 (15)	0.0442 (15)	−0.0062 (11)	−0.0052 (11)	−0.0003 (12)
C7	0.0435 (15)	0.0490 (17)	0.0440 (15)	−0.0073 (13)	−0.0059 (12)	−0.0013 (13)
C8	0.0437 (15)	0.0570 (19)	0.0474 (17)	−0.0093 (14)	−0.0067 (13)	0.0011 (14)
C9	0.0398 (14)	0.0441 (16)	0.0468 (16)	−0.0008 (12)	−0.0048 (12)	0.0003 (13)
C10	0.0418 (14)	0.0342 (14)	0.0445 (15)	0.0027 (11)	−0.0062 (12)	0.0009 (12)
C11	0.0460 (16)	0.0496 (17)	0.0495 (17)	−0.0009 (13)	−0.0117 (13)	0.0032 (13)
C12	0.065 (2)	0.071 (2)	0.0397 (16)	−0.0030 (17)	−0.0085 (14)	0.0045 (15)
C13	0.0530 (18)	0.060 (2)	0.0490 (18)	0.0006 (15)	0.0050 (14)	0.0107 (15)
C14	0.0425 (15)	0.0476 (17)	0.0597 (19)	−0.0055 (13)	−0.0049 (13)	0.0022 (14)
C15	0.0478 (16)	0.0470 (17)	0.0424 (15)	−0.0023 (13)	−0.0071 (12)	−0.0008 (13)

### Geometric parameters (Å, º)

**Table:**

Br1—C4	1.903 (3)	C8—C9	1.327 (4)
F1—C13	1.358 (4)	C8—H8A	0.9300
O1—C7	1.221 (4)	C9—C10	1.465 (4)
C1—C2	1.387 (5)	C9—H9A	0.9300
C1—C6	1.397 (4)	C10—C11	1.390 (4)
C1—H1A	0.9300	C10—C15	1.398 (4)
C2—C3	1.371 (5)	C11—C12	1.379 (5)
C2—H2A	0.9300	C11—H11A	0.9300
C3—C4	1.384 (4)	C12—C13	1.364 (5)
C3—H3A	0.9300	C12—H12A	0.9300
C4—C5	1.382 (4)	C13—C14	1.368 (5)
C5—C6	1.396 (4)	C14—C15	1.378 (4)
C5—H5A	0.9300	C14—H14A	0.9300
C6—C7	1.496 (4)	C15—H15A	0.9300
C7—C8	1.474 (4)		
			
C2—C1—C6	119.6 (3)	C7—C8—H8A	119.1
C2—C1—H1A	120.2	C8—C9—C10	127.1 (3)
C6—C1—H1A	120.2	C8—C9—H9A	116.5
C3—C2—C1	121.2 (3)	C10—C9—H9A	116.5
C3—C2—H2A	119.4	C11—C10—C15	118.2 (3)
C1—C2—H2A	119.4	C11—C10—C9	119.2 (3)
C2—C3—C4	118.6 (3)	C15—C10—C9	122.5 (3)
C2—C3—H3A	120.7	C12—C11—C10	121.3 (3)
C4—C3—H3A	120.7	C12—C11—H11A	119.3
C5—C4—C3	122.2 (3)	C10—C11—H11A	119.3
C5—C4—Br1	119.3 (2)	C13—C12—C11	118.3 (3)
C3—C4—Br1	118.5 (2)	C13—C12—H12A	120.9
C4—C5—C6	118.6 (3)	C11—C12—H12A	120.9
C4—C5—H5A	120.7	F1—C13—C12	119.0 (3)
C6—C5—H5A	120.7	F1—C13—C14	118.2 (3)
C5—C6—C1	119.8 (3)	C12—C13—C14	122.8 (3)
C5—C6—C7	118.6 (3)	C13—C14—C15	118.6 (3)
C1—C6—C7	121.5 (3)	C13—C14—H14A	120.7
O1—C7—C8	122.3 (3)	C15—C14—H14A	120.7
O1—C7—C6	120.2 (3)	C14—C15—C10	120.7 (3)
C8—C7—C6	117.5 (2)	C14—C15—H15A	119.6
C9—C8—C7	121.9 (3)	C10—C15—H15A	119.6
C9—C8—H8A	119.1		
			
C6—C1—C2—C3	1.5 (5)	C6—C7—C8—C9	−166.9 (3)
C1—C2—C3—C4	−1.4 (5)	C7—C8—C9—C10	−178.2 (3)
C2—C3—C4—C5	0.3 (5)	C8—C9—C10—C11	−165.8 (3)
C2—C3—C4—Br1	−179.2 (3)	C8—C9—C10—C15	14.1 (5)
C3—C4—C5—C6	0.7 (5)	C15—C10—C11—C12	−0.7 (5)
Br1—C4—C5—C6	−179.9 (2)	C9—C10—C11—C12	179.2 (3)
C4—C5—C6—C1	−0.6 (4)	C10—C11—C12—C13	0.3 (5)
C4—C5—C6—C7	−179.0 (3)	C11—C12—C13—F1	179.3 (3)
C2—C1—C6—C5	−0.5 (4)	C11—C12—C13—C14	1.2 (6)
C2—C1—C6—C7	177.9 (3)	F1—C13—C14—C15	179.7 (3)
C5—C6—C7—O1	25.1 (4)	C12—C13—C14—C15	−2.1 (5)
C1—C6—C7—O1	−153.3 (3)	C13—C14—C15—C10	1.7 (5)
C5—C6—C7—C8	−154.0 (3)	C11—C10—C15—C14	−0.3 (5)
C1—C6—C7—C8	27.6 (4)	C9—C10—C15—C14	179.8 (3)
O1—C7—C8—C9	14.0 (5)		

### Hydrogen-bond geometry (Å, º)

*Cg*1 and *Cg*2 are the centroids of the 3-bromophenyl (C1–C6) and 4-fluorophenyl (C10–C15) rings, respectively.

**Table:**

*D*—H···*A*	*D*—H	H···*A*	*D*···*A*	*D*—H···*A*
C9—H9*A*···O1	0.93	2.53	2.840 (4)	100
C2—H2*A*···*Cg*2^i^	0.93	2.93	3.571 (4)	127
C5—H5*A*···*Cg*2^ii^	0.93	2.98	3.642 (3)	129
C14—H14*A*···*Cg*1^iii^	0.93	2.90	3.590 (3)	132

Symmetry codes: (i) −*x*+1, −*y*+1, −*z*+1; (ii) −*x*+2, −*y*, −*z*+1; (iii) −*x*+1, −*y*, −*z*+1.
